# New Quantum Private Comparison Using Bell States

**DOI:** 10.3390/e26080682

**Published:** 2024-08-13

**Authors:** Min Hou, Yue Wu

**Affiliations:** 1 School of Computer Science, Sichuan University Jinjiang College, Meishan 620860, China; ywu@uestc.edu.cn; 2 Network and Data Security Key Laboratory of Sichuan Province, University of Electronic Science and Technology of China, Chengdu 610054, China

**Keywords:** quantum private comparison, quantum entanglement, Bell state, local operation, quantum cryptography

## Abstract

Quantum private comparison (QPC) represents a cryptographic approach that enables two parties to determine whether their confidential data are equivalent, without disclosing the actual values. Most existing QPC protocols utilizing single photons or Bell states are considered highly feasible, but they suffer from inefficiency. To address this issue, we present a novel QPC protocol that capitalizes on the entanglement property of Bell states and local operations to meet the requirements of efficiency. In the proposed protocol, two participants with private inputs perform local operations on shared Bell states received from a semi-honest third party (STP). Afterward, the modified qubits are returned to the STP, who can then determine the equality of the private inputs and relay the results to the participants. A simulation on the IBM Quantum Cloud Platform confirmed the feasibility of our protocol, and a security analysis further demonstrated that the STP and both participants were unable to learn anything about the individual private inputs. In comparison to other QPC protocols, our proposed solution offers superior performance in terms of efficiency.

## 1. Introduction

The advancement of quantum technology has rendered classical cryptographic algorithms such as RSA and ElGamal, which rely on the difficulty of factoring large numbers, vulnerable to quantum algorithm attacks such as the Shor algorithm [1]. Quantum cryptography, which integrates classical cryptography and quantum mechanics, provides unconditional security by leveraging quantum properties. Consequently, quantum cryptography has garnered considerable attention from the academic community. The pioneering BB84 protocol, proposed by Bennett and Brassard in 1984 [2], enhanced information transmission security and privacy. Since then, numerous quantum cryptographic protocols have emerged, targeting objectives like quantum key distribution (QKD) [3,4,5,6], quantum key agreement (QKA) [7,8,9], quantum secret sharing (QSS) [10,11,12], quantum secure direct communication (QSDC) [13,14], and quantum private set intersection [15].

Private comparison originated from Yao’s millionaires’ problem [16], which aims to determine the richer of two millionaires while keeping their wealth undisclosed. The socialist millionaires’ problem, a variation of Yao’s millionaires’ problem, aims to determine whether two individuals have equal wealth, as proposed by Boudot et al. [17]. Since then, solving the millionaires’ problem has become a fundamental task in the field of secure multiparty computing (SMC). When addressing this task, Lo [18] pointed out that designing a protocol to securely evaluate a two-party computational function is impossible. Consequently, the involvement of a semi-honest third party (STP) should be considered when developing private comparison protocols. The security of private comparison protocols is similar to classical cryptography, which relies on the difficulty of factorizing large numbers and is consequently vulnerable to quantum attacks. As a result, new measures need to be implemented to achieve quantum security.

Quantum private comparison (QPC) differentiates itself from classical private comparison by employing qubits as quantum information carriers, rather than classical bits. The goal of QPC is to compare the equality of two participants’ private inputs while preserving the privacy of both parties and ensuring quantum-based security. In 2009, Yang and Wen [19] introduced the first QPC protocol, utilizing the property of Bell states to achieve private comparison and decoy photons to detect the presence of an eavesdropper. In 2010, Chen et al. [20] introduced a new QPC protocol by performing unitary operations on GHZ states to encode information, but it could not defend against interception attacks [21]. Since these early contributions, the field of QPC has seen a proliferation of proposed protocols, each exploring the use of diverse quantum states as information carriers. The current landscape of QPC protocols can be broadly categorized into two main streams:

➢ QPC protocols using low-dimensional quantum states;
➢ QPC protocols using high-dimensional quantum states.

Single photons, Bell states, GHZ states [22], and W states are commonly employed as quantum resources to achieve private comparison in low-dimensional quantum state-based QPC protocols. Huang et al. [23] utilized single photons and collective detection to design a QPC protocol, where a specific unitary operation was performed on single photons to encode information. However, this protocol consumed 4n qubits to compare n classical bits, resulting in a qubit efficiency of only 25%. Li et al. [24] introduced a novel protocol that leveraged the concept of entanglement swapping between Bell states and W-class states. This protocol enabled the comparison of two classical bits per round, achieving an efficiency of 40%. However, Gao et al. [25] argued that the STP assumption in the scheme presented in Ref. [24] is unreasonable, and that the scheme cannot withstand fake signal attacks when the STP restriction is tightened, leading to the disclosure of privacy to the STP. Lang et al. [26] utilized Bell states as quantum resources and employed quantum gates instead of classical exclusive-OR computations for classical computing, an approach that can enhance the security. However, Duan [27] noted that the scheme presented in Ref. [26] is vulnerable to measurement attacks from the STP and disturbance attacks from external adversaries, and proposed some improvements to address these security weaknesses. Huang et al. [28] introduced a novel protocol that harnessed the properties of entanglement swapping between three Bell states, enabling the comparison of three classical bits per round and achieving a qubit efficiency of 50%. Hou and Wu [29] proposed a QPC protocol that employed single photons as well as Identity or Hadamard operations to achieve private comparison. However, the need for multiple preparations of single photons as the initial quantum resources in this protocol leads to a reduction in qubit efficiency. While the low-dimensional quantum state-based QPC protocols can achieve private comparison while preserving the secrecy of the inputs, these protocols generally exhibit lower qubit efficiency.

In high-dimensional quantum state-based QPC protocols, quantum states with a large number of degrees of freedom or a large Hilbert space dimension are primarily employed as quantum resources to enable private comparison. Jia et al. [30] proposed a QPC protocol that utilizes d-level GHZ states as the quantum resources. In this protocol, the secrets are encoded into the phase of the d-level GHZ states through local operations, and the phase information can then be collectively measured to facilitate the private comparison. Lin et al. [31] designed a QPC protocol that employed d-dimensional Bell states as the quantum resources. Through the strategic use of unitary operations, the secrets are encoded, enabling the comparison of the size relationship of the private inputs. Guo et al. [32] employed entanglement swapping between d-level states to achieve the comparison and shifting operations to encode the private inputs of the participants. Recognizing the importance of practical implementation, Yu et al. [33] developed a QPC protocol that utilized easy-to-implement d-level single-particle states to compare the size relationship of the private inputs. Aiming to enhance capacity and reduce the quantum resource requirements, Xu and Zhao [34] proposed a QPC protocol that employed Bell states as the quantum resources, achieving a significantly higher capacity compared to previous approaches. Ji et al. [35] introduced a QPC protocol that employed (n + 1)-qubit GHZ states, where n represents the number of qubits in the GHZ states, ensuring the privacy of the participants by generating a secret key and performing bit-flipping operations. Wu and Zhao [36] utilized d-level Bell states for private comparison to determine the relationship of the private inputs. While quantum state-based QPC protocols can encode more quantum information and achieve private comparison while maintaining secrecy, they pose significant challenges in practical implementation with current technology due to the difficulty of preparing complex quantum states.

An analysis of the existing QPC protocols revealed that those utilizing low-dimensional quantum states have lower qubit efficiency, while those employing high-dimensional quantum states pose significant challenges in practical implementation with the current technology. To address this issue, we propose a QPC protocol employs Bell states and local operations to facilitate private comparison. Specifically, the inputs are encoded into shared Bell states, which are then sent to the semi-trusted party (STP). The STP can subsequently determine the equality of the inputs without learning the individual values and communicate the results back to the participants. Simulation experiments conducted on the IBM Quantum Cloud Platform have demonstrated the practical viability of this approach. Additionally, the protocol’s security analysis suggests its ability to withstand both outsider attacks from eavesdroppers and participant attacks aimed at learning the individual inputs. Compared to other QPC protocols, the proposed solution utilizes a Bell state, which is relatively straightforward to implement, to compare a single classical bit. This results in a notable qubit efficiency of 50%.

The remaining sections are organized as follows. Section 2 provides some preliminaries, introducing the necessary background information. Section 3 then presents the detailed steps of the designed QPC protocol. Simulations and security analyses are discussed in Section 4 and Section 5, respectively. Section 6 includes an efficiency analysis and comparison. Finally, Section 7 summarizes the key findings of this work.

## 2. Preliminaries

The bit flip and phase shift operators can be given by

(1) $$X=\left(\begin{array}{cc} 1 & 0\\ 0 & 1 \end{array}\right),\;Z=\left(\begin{array}{cc} 1 & 0\\ 0 & -1 \end{array}\right)$$

Applying the above two operators to an orthonormal basis $\left\{\left|0\right\rangle ,\left|1\right\rangle \right\}$, we have

(2) $$\left\{\begin{array}{c} X|0\rangle =|1\rangle ,X|1\rangle =|0\rangle \\ Z|0\rangle =|0\rangle ,Z|1\rangle =-|1\rangle \end{array}\right.$$

Four Bell states can be written as

(3) $$|\varphi_{00}\rangle =\frac{1}{\sqrt{2}}\left(|00\rangle +|11\rangle \right)$$

(4) $$|\varphi_{01}\rangle =\frac{1}{\sqrt{2}}\left(|00\rangle -|11\rangle \right)$$

(5) $$|\varphi_{10}\rangle =\frac{1}{\sqrt{2}}\left(|01\rangle +|10\rangle \right)$$

(6) $$|\varphi_{11}\rangle =\frac{1}{\sqrt{2}}\left(|01\rangle -|10\rangle \right)$$

Suppose that a third party prepares a Bell state $\left|\varphi_{ab}\right\rangle$ and distributes the first qubit to Alice and the second qubit to Bob. Subsequently, Alice and Bob perform the following operations: if a=0, Alice applies the phase shift operator *Z* to her qubit; if a=1, Alice applies the bit flip operator X to her qubit. Likewise, if b=0, Bob applies the phase shift operator Z to his qubit, and if b=1, Bob applies the bit flip operator X to his qubit. The encoding rule is presented in Table 1. After Alice and Bob have applied their respective operations, they return their individual qubits to the third party. The third party then performs a Bell basis measurement on the combined state, obtaining the measurement result $\left|\varphi_{ab}^{\prime }\right\rangle$. The resulting states when performing the bit flip and phase shift operators on $\left|\varphi_{ab}\right\rangle$ are shown in Table 2.

## 3. Quantum Private Comparison Protocol

The QPC protocol involves three entities.

**Semi-honest third party (STP)**: STP has full quantum capabilities and operates in the semi-honest model. In the semi-honest model, the STP must strictly follow the defined protocol steps but may attempt to learn the users’ secrets by utilizing the immediate results and performing quantum attacks. However, the STP is not allowed to collude with or favor any of the users involved.

**Users:** Two users, Alice and Bob are involved in the protocol to compare their secrets. Like the STP, both Alice and Bob have full quantum capabilities. However, they adopt an honest-but-curious posture—adhering strictly to the established protocol, while potentially seeking to uncover each other’s secrets.

**Figure 1:**
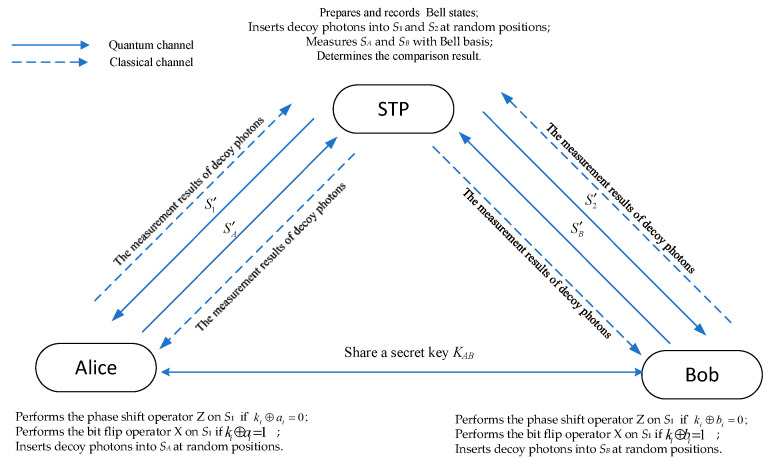
The diagram of the QPC protocol.

Participants Alice and Bob each possess their own private data, denoted as A and B, respectively. These secrets can be written in binary form as $A=\left\{a_{L-1}a_{L-2}\cdots a_{1}a_{0}\right\}$ and $B=\left\{b_{L-1}b_{L-2}\cdots b_{1}b_{0}\right\}$, where $a_{i},b_{i}\in \left\{0,1\right\},i=0,1,2,\cdots L,$ and *L* is the length of the strings A and B. The protocol assumes a quantum channel that is free from noise and loss, while the classical channel is authenticated during transmission. The detailed steps of the proposed QPC protocol are as follows, and its diagram is shown in Figure 1.

**Step 1**. Alice and Bob use a QKD protocol, such as the BB84 protocol [2], to generate a shared secret key $K_{AB}=\left(k_{L-1}k_{L-2}\cdots k_{1}k_{0}\right)$, where $k_{i}\in \left\{0,1\right\},i=0,1,2,\cdots ,L$.

**Step 2.** STP prepares *n* Bell states, all randomly chosen from Equations (3)–(6), records these Bell states, and distributes the first qubits as sequence *S*_1_ and the second qubits as sequence *S*_2_. Then, she prepares 2δ decoy photons, each chosen from $\left\{\left|0\right\rangle ,\left|1\right\rangle ,\left|+\right\rangle =\frac{\left|0\right\rangle +\left|1\right\rangle }{\sqrt{2}},\left|-\right\rangle =\frac{\left|0\right\rangle -\left|1\right\rangle }{\sqrt{2}}\right\}$ randomly, and inserts δ decoy photons into *S*_1_ and another δ decoy photons into *S*_2_ at random positions to generate two sequences, $S_{1}^{\prime }$ and $S_{2}^{\prime }$, respectively. Finally, she sends $S_{1}^{\prime }$ and $S_{2}^{\prime }$ to Alice and Bob, respectively, via the quantum channel.

**Step 3**. After receiving the respective photon sequences $S_{1}^{\prime }$ and $S_{2}^{\prime }$, Alice and Bob each send a confirmation message to the STP. In response, the STP announces the positions and measurement bases of the decoy photons within the sequences. For example, if the decoy photons are prepared in $\left|0\right\rangle$ or $\left|1\right\rangle$ states, the measurement base is the Z basis. Otherwise, the measurement basis is the X basis. Alice and Bob then measure the decoy photons using the announced bases and send the results back to the STP over a classical channel. The STP compares the initial decoy photon preparations with the measurement results to compute the error rate. If the error rate exceeds a predefined threshold, the protocol is terminated. Otherwise, the process continues to the next step.

**Step 4**. Alice (Bob) discards the decoy photons in $S_{1}^{\prime }\left(S_{2}^{\prime }\right)$ to obtain *S*_1_ and *S*_2_.

For Alice:

(1) If $k_{i}⊕a_{i}=0$, she performs the phase shift operator Z on *S*_1_. Otherwise, she performs the bit flip operator X on *S*_1_. The resulting sequence is denoted as $S_{A}$.
(2) She inserts δ decoy photons, chosen from $\left\{\left|0\right\rangle ,\left|1\right\rangle ,\left|+\right\rangle =\frac{\left|0\right\rangle +\left|1\right\rangle }{\sqrt{2}},\left|-\right\rangle =\frac{\left|0\right\rangle -\left|1\right\rangle }{\sqrt{2}}\right\},$ into $S_{A}$ to generate a new sequence $S_{A}^{\prime }$.
(3) She sends $S_{A}^{\prime }$ to the STP via a quantum channel.

For Bob:

(1) If $k_{i}⊕b_{i}=0$, he performs the phase shift operator Z on *S*_2_. Otherwise, he performs the bit flip operator X on *S*_2_. The resulting sequence is denoted as $S_{B}$.
(2) She inserts δ decoy photons, chosen from $\left\{\left|0\right\rangle ,\left|1\right\rangle ,\left|+\right\rangle =\frac{\left|0\right\rangle +\left|1\right\rangle }{\sqrt{2}},\left|-\right\rangle =\frac{\left|0\right\rangle -\left|1\right\rangle }{\sqrt{2}}\right\},$ into $S_{B}$ to generate a new sequence $S_{B}^{\prime }$.
(3) She sends $S_{B}^{\prime }$ to the STP via a quantum channel.

**Step 5**. When the STP receives $S_{A}^{\prime }$ and $S_{B}^{\prime }$, she interacts with Alice and Bob to check for the presence of an eavesdropper, similar to the process carried out in Step 3. If no eavesdropper is detected, the protocol proceeds to the next step. However, if an eavesdropper is detected, the protocol is terminated.

**Step 6**. The STP discards the decoy photons in $S_{A}^{\prime }$ and $S_{B}^{\prime }$ to obtain $S_{A}$ and $S_{B}$ and then performs the Bell measurement on them. If the Bell measurement results match the initially prepared Bell states, the STP can conclude that the inputs provided by Alice and Bob are identical. Conversely, if the Bell measurement results differ from the initial Bell states, the STP determines that the inputs from Alice and Bob are different. 

## 4. Simulation Experiments

**Figure 2:**
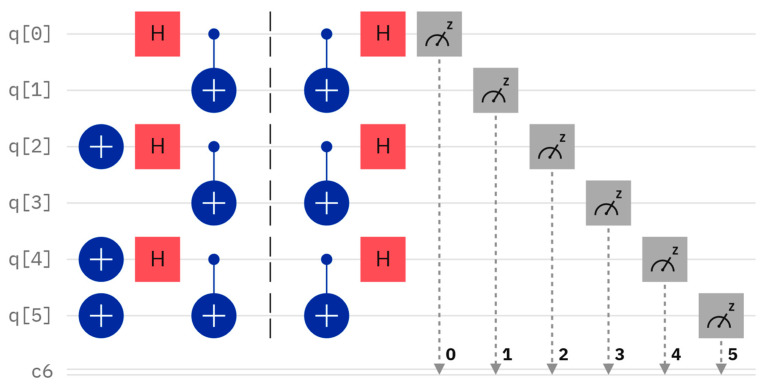
Preparation of Bell states.

**Figure 3:**
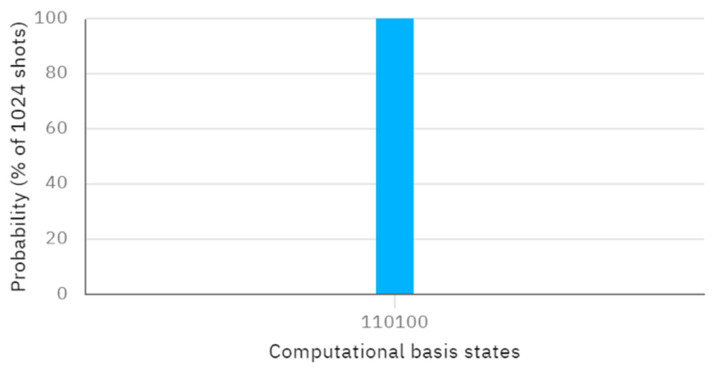
Measurement results of the Bell states.

Considering a concrete example, let us assume that Alice and Bob have their own secrets, A = 7 and B = 6, with their binary representations being A=111 and B=110, respectively. Since the length of both A and B is 3, three Bell states are assumed to be prepared as $\left|\varphi_{00}\right\rangle ,\left|\varphi_{01}\right\rangle$, and $\left|\varphi_{11}\right\rangle$. The quantum circuit and measurement result are shown in Figure 2 and Figure 3, respectively. For example, the Bell state $\left|\varphi_{00}\right\rangle$ can be produced using a Hadamard gate and a controlled-NOT gate, which is performed on two qubits corresponding to q [0] and q [1] of Figure 2, starting from the state $\left|0\right\rangle$. To measure the Bell state $\left|\varphi_{00}\right\rangle$, we can perform the Hadamard gate and the controlled-NOT gate once, and the measurement result will always be $\left|00\right\rangle$, corresponding to 00. For the other three Bell states $\left|\varphi_{01}\right\rangle ,\left|\varphi_{10}\right\rangle ,\;and\;\left|\varphi_{11}\right\rangle$, their measurement outcomes using the Bell measurement will corresponding to 10, 01, and 11, respectively.

**Figure 4:**
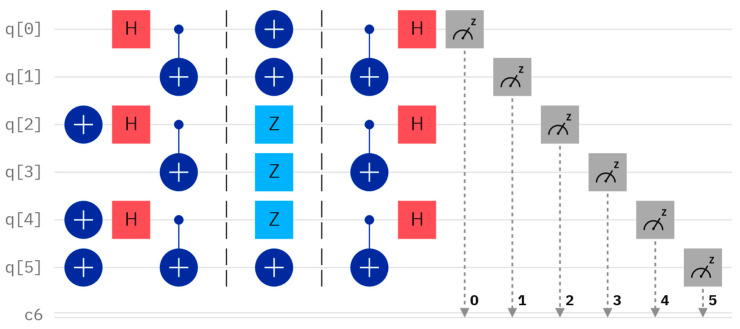
Quantum circuit for comparing *A* and *B.*

**Figure 5:**
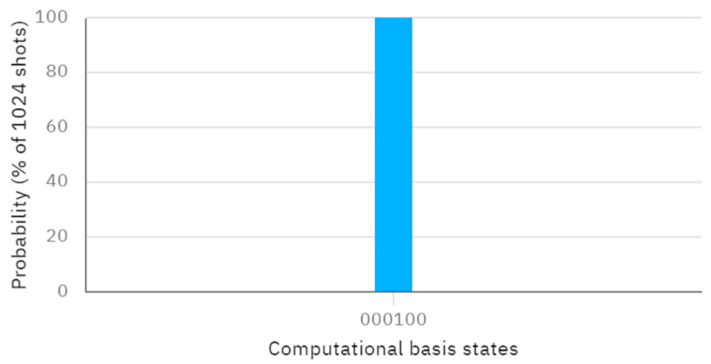
The measurement results of Figure 4.

Assume a secret key $K_{AB}=011$ of length L is shared. According to this protocol, when Alice performs X, Z, and Z operators on the first qubit of $\left|\varphi_{00}\right\rangle ,\left|\varphi_{01}\right\rangle ,$ and $\left|\varphi_{11}\right\rangle$, and Bob performs X, Z, and X operators on the second qubit of $\left|\varphi_{00}\right\rangle ,\left|\varphi_{01}\right\rangle ,$ and $\left|\varphi_{11}\right\rangle$, the resulting quantum circuit without considering the eavesdropping detection, which can be considered as an independent procedure, and the corresponding measurement outcome, obtained through Bell measurement, are depicted in Figure 4 and Figure 5, respectively. Examining Figure 5, the measured outcome of the quantum circuit in Figure 4 is 001000, corresponding to q [0]–q [5]. This differs from the measurement result of 001011, corresponding to q [0]–q [5], as shown in Figure 3. The discrepancy between the actual and expected measurement outcomes suggests that the measurement results do not match the initially prepared Bell states. This indicates that the inputs provided by Alice and Bob are not identical (i.e., the comparison result is A≠B). 

**Figure 6:**
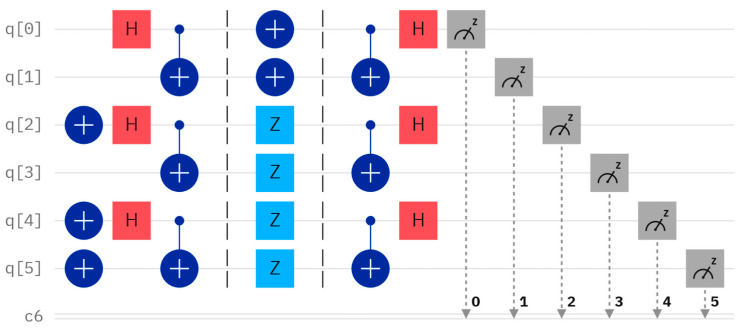
Quantum circuit for comparing $A^{\prime }\;and\;B^{\prime }$.

**Figure 7:**
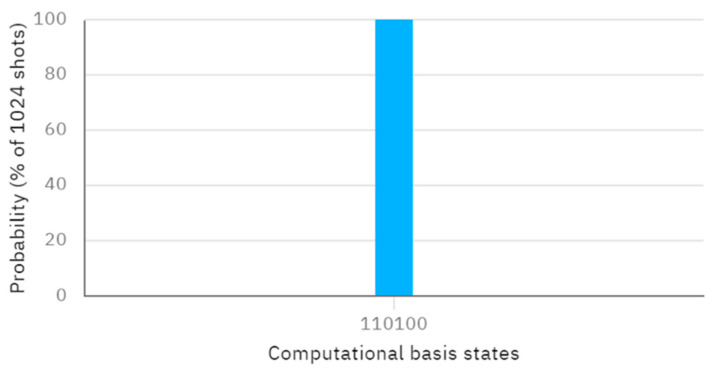
The measurement result of Figure 6.

Considering another concrete case, let us assume that Alice and Bob have their own secrets, $A^{\prime }=7$ and $B^{\prime }=7$, with their binary representations being $A^{\prime }=111$ and $B^{\prime }=111$, respectively. The initially-prepared Bell states and $K_{AB}$ are the same as the previous assumptions. According to this protocol, when Alice performs X, Z, and Z operators on the first qubit of $\left|\varphi_{00}\right\rangle ,\left|\varphi_{01}\right\rangle ,$ and $\left|\varphi_{11}\right\rangle$, and Bob performs X, Z, and Z operators on the second qubit of $\left|\varphi_{00}\right\rangle ,\left|\varphi_{01}\right\rangle ,$ and $\left|\varphi_{11}\right\rangle$, The resulting quantum circuit and the corresponding measurement outcome, obtained through Bell measurement, are depicted in Figure 6 and Figure 7, respectively. Examining Figure 7, the measured outcome of the quantum circuit in Figure 6 is 001011, corresponding to q [0]–q [5]. This is consistent with the measurement result of 001011, as shown in Figure 3. The fact that the actual and expected measurement outcomes match indicates that the measurement results are the same as the initially prepared Bell states. This suggests that the inputs provided by Alice and Bob are identical (i.e., the comparison result is $A^{\prime }=B^{\prime }$).

To conclude, the above two concrete cases reveal the feasibility of our protocol.

## 5. Security Analysis

During the transmission of quantum information, external attackers may eavesdrop on the quantum channel to steal useful information about the inputs of the users. Additionally, the users themselves may try to utilize some received immediate results to deduce the private inputs of another user. However, our protocol is designed to resist both eavesdropping and participant attacks. This is achieved through the decoy state method and quantum key distribution (QKD) techniques employed in the protocol.

On the one hand, the protocol can detect the presence of eavesdroppers by randomly inserting decoy states, which are states with known properties, into the transmitted signal. Any tampering or eavesdropping on the quantum channel can be detected by analyzing the properties of the received decoy states. On the other hand, the protocol utilizes QKD to establish a shared secret key between the participating users. This shared key is then used to covert the transmitted information, ensuring that even if an attacker manages to intercept the quantum signals, they will not be able to extract any meaningful information without the secret key. By employing these techniques, the proposed protocol is able to effectively mitigate the risks of both external eavesdropping and participant attacks, thereby ensuring the security and integrity of the transmitted quantum information.

### 5.1. External Attacks

Potential attacker, Eve, may attempt diverse classical or quantum-based assaults to acquire information about the user inputs. Possible attacks encompass intercept-measure-resend, entangle-measure, and Trojan horse techniques [37,38,39,40]. Nonetheless, the proposed protocol leverages the decoy state method during qubit transmission between parties. This approach effectively counters these varied attack vectors.

#### 5.1.1. Intercept-Measure-Resend Attack

In an intercept-measure-resend attack, the adversary (Eve) intercepts the quantum channel, measures the intercepted sequence using guessed bases, and resends a new sequence with the same measurement results. However, this would trigger protocol termination, as the computed error rate exceeds the threshold. Eve lacks knowledge to distinguish decoy photons from target particles, and the decoy photon measurement bases are unknown to her. Eve may attempt to use guessed bases to obtain some information. For a decoy photon, there is a 50% chance of correctly guessing the base. Similarly, choosing the wrong base can still bypass detection half the time. This means that Eve can bypass detection with a 25% probability when using the wrong base. For example, measuring a |+⟩ decoy photon using the Z-basis results in $\left|0\right\rangle$ or $\left|1\right\rangle$ states. When preparing $\left|0\right\rangle$ or $\left|1\right\rangle$ and sending them to the receiver, Eve will not introduce any error with a probability of 50%. Measuring the decoy with the X-basis gives $\left|+\right\rangle$, which can be sent without error, bypassing detection with 100% probability. Overall, the probability that Eve can intercept a decoy photon without introducing error and bypass detection is 0.75.

In summary, the probability of detecting the adversary (Eve) during the detection process is given by $1-{\left(\frac{3}{4}\right)}^{\delta }$, where δ is the number of decoy photons. When δ = 27, the probability of detecting Eve is 0.9996, and as δ increases further, the probability of detecting Eve tends toward 1. Consequently, when an adversary performs the intercept-measure-resend attack, they will inevitably introduce errors into the transmitted sequence, which can then be detected by the legitimate users. Due to the high probability of detection and the introduction of errors, the adversary cannot learn anything about the inputs of the users during the QPC process.

#### 5.1.2. Entangle-Measure Attack

The entangle-measure attack refers to a strategy where Eve performs an interception operation on the quantum channel to obtain the transmitted quantum sequence. Eve then prepares auxiliary particles $\left|e\right\rangle$ and entangles them with each intercepted particle, with the aim of stealing the secrets of the users. When the target particles are measured, Eve can measure the auxiliary particles to learn the states of the target particles. However, the success of this attack is contingent on Eve being able to avoid detection by the eavesdropping detection mechanism.

When entangling the auxiliary particle $\left|e\right\rangle$ with a target particle in state $\left|0\right\rangle$ or $\left|1\right\rangle$, we have the following equations:

(7) $$U\left|e\right\rangle \left|0\right\rangle =\lambda_{0}\left|0e_{00}\right\rangle +\lambda_{1}\left|0e_{01}\right\rangle$$

(8) $$U\left|e\right\rangle \left|1\right\rangle =\lambda_{2}\left|0e_{10}\right\rangle +\lambda_{3}\left|0e_{11}\right\rangle$$

where the parameters $\lambda_{0}$, $\lambda_{1}$, $\lambda_{2}$, and $\lambda_{3}$ satisfy ${\left\|\lambda_{0}\right\|}^{2}+{\left\|\lambda_{1}\right\|}^{2}={\left\|\lambda_{2}\right\|}^{2}+{\left\|\lambda_{3}\right\|}^{2}=1$. Four quantum states $\left\{\left|e_{00}\right\rangle ,\left|e_{01}\right\rangle ,\left|e_{10}\right\rangle ,\left|e_{11}\right\rangle \right\}$ are determined by U.

When entangling the auxiliary particle $\left|e\right\rangle$ with a target particle in state $\left|+\right\rangle$ or $\left|-\right\rangle$, the resultant process can be written as:

(9) $$\begin{array}{l} U|e\rangle |+\rangle =\frac{1}{\sqrt{2}}\left(\lambda_{0}|0e_{00}\rangle +\lambda_{1}|1e_{01}\rangle +\lambda_{2}|0e_{10}\rangle +\lambda_{3}|1e_{11}\rangle \right)\\ =\frac{1}{2}|+\rangle \left(\lambda_{0}|e_{00}\rangle +\lambda_{1}|e_{01}\rangle +\lambda_{2}|e_{10}\rangle +\lambda_{3}|e_{11}\rangle \right)\\ +\frac{1}{2}|-\rangle \left(\lambda_{0}|e_{00}\rangle -\lambda_{1}|e_{01}\rangle +\lambda_{2}|e_{10}\rangle -\lambda_{3}|e_{11}\rangle \right) \end{array}$$

(10) $$\begin{array}{l} U|e\rangle |-\rangle =\frac{1}{\sqrt{2}}\left(\lambda_{0}|0e_{00}\rangle +\lambda_{1}|1e_{01}\rangle -\lambda_{2}|0e_{10}\rangle -\lambda_{3}|1e_{11}\rangle \right)\\ =\frac{1}{2}|+\rangle \left(\lambda_{0}|e_{00}\rangle +\lambda_{1}|e_{01}\rangle -\lambda_{2}|e_{10}\rangle -\lambda_{3}|e_{11}\rangle \right)\\ +\frac{1}{2}|-\rangle \left(\lambda_{0}|e_{00}\rangle -\lambda_{1}|e_{01}\rangle -\lambda_{2}|e_{10}\rangle +\lambda_{3}|e_{11}\rangle \right) \end{array}$$

To introduce no error, the above Equations (7)–(10) should meet the following conditions:

(11) $$\lambda_{1}=\lambda_{2}=0$$

(12) $$\lambda_{0}=\lambda_{3}=1$$

(13) $$\lambda_{0}|e_{00}\rangle -\lambda_{1}|e_{01}\rangle +\lambda_{2}|e_{10}\rangle -\lambda_{3}|e_{11}\rangle =\vec{0}$$

(14) $$\lambda_{0}|e_{00}\rangle +\lambda_{1}|e_{01}\rangle -\lambda_{2}|e_{10}\rangle -\lambda_{3}|e_{11}\rangle =\vec{0}$$

From Equations (11)–(14), we can further obtain $\lambda_{0}=\lambda_{3}=1$ and $\left|e_{00}\right\rangle =\left|e_{11}\right\rangle$. When substituting these results into Equations (7)–(10), we have

(15) $$U|e\rangle |0\rangle =|0e_{00}\rangle =|0e_{11}\rangle$$

(16) $$U|e\rangle |1\rangle =|1e_{00}\rangle =|1e_{11}\rangle$$

(17) $$U|e\rangle |+\rangle =|+e_{00}\rangle =|+e_{11}\rangle$$

(18) $$U|e\rangle |-\rangle =|-e_{00}\rangle =|-e_{11}\rangle$$

We can conclude that the auxiliary and target particles exist in a product form, rather than a tensor product. This independent relationship between the auxiliary and target particles indicates an absence of entanglement. Furthermore, when the target particles are measured, Eve cannot know the states of the target particles when performing the measurement on the auxiliary particles. As a result, Eve cannot infer the secrets by conducting the entangle-measure attack.

#### 5.1.3. Trojan Horse Attack

Two-way quantum communication protocols are susceptible to Trojan horse attacks, which encompass the delay-photon Trojan-horse attack and the invisible photon eavesdropping Trojan-horse attack [41]. Given that the proposed protocol is a two-way quantum communication protocol, it may be vulnerable to such attacks. However, several existing techniques can be employed to detect and mitigate Trojan horse attacks. For example, the wavelength quantum filter (WQF) and the photons number splitter (PNS) can be utilized to eliminate invisible photons and separate legitimate photons when encountering Trojan horse attacks.

### 5.2. Participant Attacks

Insider participants may launch more powerful attacks to steal the secrets. The following discusses two cases of attacks from the semi-honest third party (STP) and Alice or Bob.

#### 5.2.1. Attacks from the STP

In the semi-honest model, the STP is involved and must strictly follow the defined steps of the protocol, but is not allowed to collude or favor any of the users. STP may attempt to learn the users’ secrets by utilizing the immediate results and performing quantum attacks. In the proposed protocol, the STP is primarily involved in preparing Bell states and performing Bell-basis measurement. Although the STP knows the initially-prepared Bell state and the final Bell states, it cannot infer the secrets by utilizing the relationship between them. On the one hand, the STP may prepare a single-photon sequence to replace the Bell state sequence and measure the final received quantum sequence with single-particle measurement. For example, suppose that the STP prepares a single photon in state $\left|1\right\rangle$, which is sent to Alice. The final measurement result is $\left|1\right\rangle$ when the Z operator is performed by Alice, and the final measurement result is $\left|0\right\rangle$ when the X operator is performed by Alice. Therefore, regardless of the Z or X operators performed by Alice, the STP can know them by measuring the received quantum sequence with single-particle measurement. However, the STP has the opportunity to know which operations are performed by Alice and Bob, but has no way of knowing the secret key, since *K_AB_* is unknown to her, making it impossible to eavesdrop on the secrets. On the other hand, the STP may launch attacks that are similar to the malicious behavior performed by Eve, but these will be inevitably detected, as discussed in Section 5.1. As a result, the STP has no way of learning the secrets.

#### 5.2.2. Attacks from Alice or Bob

The proposed protocol assumes the same roles for the participants, Alice and Bob, without loss of generality. The protocol presumes that Bob seeks to ascertain Alice’s secrets, which are encoded into quantum operations, such as bit flip and phase shift, used to transform the received quantum sequence $S_{1}$. If Bob intends to know the secrets of Alice, he must know the converted quantum sequence $S_{A}$ and the initially prepared Bell state sequence. However, there is no direct communication between the participants; the only way for Bob to obtain $S_{1}$ and $S_{A}$ is to intercept the quantum communication between the STP and Alice. This behavior is equivalent to performing external eavesdropping and will inevitably be detected due to the decoy state method employed. A similar method can be used to analyze the situation where Alice intends to know the secrets of Bob. Consequently, the secrets of both participants will remain undisclosed, even in the event of participant attacks.

## 6. Efficiency Analysis and Comparison

The qubit efficiency [42] is a crucial metric for evaluating the efficiency of a quantum communication protocol, which can be expressed as:

(19) $$e=\frac{c}{t}$$

where *e* represents the qubit efficiency, c denotes the number of classical bits compared, and *t* represents the total number of qubits consumed during the entire process excluding the decoy photons, which can be considered as an independent procedure. In the protocol, a Bell state with two qubits can be used to compare one classical bit, and therefore, *c* = *L* and *t* = 2*L*. Therefore, the qubit efficiency of the protocol is 50%.

Table 3 presents a comparison of the proposed QPC protocol with some existing QPC protocols in terms of quantum resource, entanglement swapping, unitary operation, quantum measurement, and qubit efficiency.

The quantum resource usage and qubit efficiency of the proposed protocol are the same as the protocol presented in Ref [28], as demonstrated in Table 3. However, the unitary operations, such as bit flip and phase shift, employed in the proposed protocol are comparatively more straightforward to implement than the entanglement swapping and GHZ-basis measurements required in Ref [28]. While the quantum resources in Refs. [19,20,22,23,28] and the proposed protocol are easy to implement, the qubit efficiency in Refs. [19,20,22,23] is relatively low. Therefore, the proposed QPC protocol not only has a higher qubit efficiency, but also offers easier implementation due to the simpler unitary operations required, resulting in an overall superior performance.

## 7. Conclusions

In this paper, we proposed a novel QPC protocol that harnesses the entanglement property of Bell states to enable the comparison of private inputs. The proposed protocol employs a readily implementable Bell state to compare a single classical bit, thereby achieving a qubit efficiency of 50%. Furthermore, the protocol’s architecture, which is grounded in the utilization of Bell states, unitary operations, and Bell measurements, confers greater practicality and feasibility. We conducted the simulation experiments on the IBM Quantum Cloud Platform, which validated the feasibility of the proposed protocol. Furthermore, our security analysis substantiates the proposed protocol’s ability to effectively safeguard the participants’ secrets from both external eavesdropping and participant-based attacks.

## Figures and Tables

**Table 1:** The encoding rule.

	a=0	a=1
b=0	Z⊗Z	X⊗Z
b=1	Z⊗X	X⊗X

**Table 2:** The resulting states when performing the encoding rule on $|\varphi_{00}\rangle$, $|\varphi_{01}\rangle$, $|\varphi_{10}\rangle$, and $|\varphi_{11}\rangle$.

	a=0,b=0	a=0,b=1	a=1,b=0	a=1,b=1
$|\varphi_{00}\rangle$	$|\varphi_{00}\rangle$	$|\varphi_{11}\rangle$	$|\varphi_{11}\rangle$	$|\varphi_{00}\rangle$
$|\varphi_{01}\rangle$	$|\varphi_{01}\rangle$	$|\varphi_{10}\rangle$	$|\varphi_{10}\rangle$	$|\varphi_{01}\rangle$
$|\varphi_{10}\rangle$	$|\varphi_{10}\rangle$	$|\varphi_{01}\rangle$	$|\varphi_{01}\rangle$	$|\varphi_{10}\rangle$
$|\varphi_{11}\rangle$	$|\varphi_{11}\rangle$	$|\varphi_{00}\rangle$	$|\varphi_{00}\rangle$	$|\varphi_{11}\rangle$

**Table 3:** Comparison of our protocol among some of the existing QPC protocols.

	Ref. [19]	Ref. [20]	Ref. [22]	Ref. [23]	Ref. [28]	Ours
Quantum resource	Bell states	GHZ states	GHZ states	Single photons	Bell states	Bell states
Entanglement swapping	No	No	Yes	No	Yes	No
Unitary operation	Yes	Yes	No	Yes	No	Yes
Quantum measurement	Bell basis	Single-particle	Bell basis	Single-particle	GHZ basis	Bell basis
Qubit efficiency	25%	33%	33%	25%	50%	50%

## Data Availability

No new data were created or analyzed in this study. Data sharing is not applicable to this article.
